# A Real-Time PCR Screening Assay for Rapid Detection of *Listeria Monocytogenes* Outbreak Strains

**DOI:** 10.3390/foods9010067

**Published:** 2020-01-08

**Authors:** Marina Torresi, Anna Ruolo, Vicdalia Aniela Acciari, Massimo Ancora, Giuliana Blasi, Cesare Cammà, Patrizia Centorame, Gabriella Centorotola, Valentina Curini, Fabrizia Guidi, Maurilia Marcacci, Massimiliano Orsini, Francesco Pomilio, Marco Di Domenico

**Affiliations:** 1 Istituto Zooprofilattico Sperimentale dell’Abruzzo e del Molise G. Caporale, via Campo Boario, 64100 Teramo TE, Italy; 2 Istituto Zooprofilattico Sperimentale dell’Umbria e delle Marche Togo Rosati, Via Gaetano Salvemini, 1, 06126 Perugia PG, Italy; 3 Istituto Zooprofilattico Sperimentale delle Venezie, Viale dell’Università, 10, 35020 Legnaro PD, Italy

**Keywords:** *Listeria monocytogenes*, outbreak, molecular methods, real-time PCR, screening

## Abstract

From January 2015 to March 2016, an outbreak of 23 human cases of listeriosis in the Marche region and one human case in the Umbria region of Italy was caused by *Listeria monocytogenes* strains showing a new pulsotype never described before in Italy. A total of 37 clinical strains isolated from patients exhibiting listeriosis symptoms and 1374 strains correlated to the outbreak were received by the Italian National Reference Laboratory for *L. monocytogenes* (It NRL Lm) of Istituto Zooprofilattico Sperimentale dell’Abruzzo e del Molise (IZSAM) for outbreak investigation. A real-time PCR assay was purposely designed for a rapid screening of the strains related to the outbreak. PCR-positive strains were successively typed through molecular serogrouping, pulsed field gel electrophoresis (PFGE), and Next Generation Sequencing (NGS). Applying the described strategy, based on real-time PCR screening, we were able to considerably reduce time and costs during the outbreak investigation activities.

## 1. Introduction

Listeriosis is a foodborne illness caused by *Listeria monocytogenes*. The most common symptoms of the mild form of the disease include diarrhoea, fever, headache, and myalgia. However, when listeriosis appears as an invasive infection, patients may develop more severe outcomes such as meningitis and/or septicemia in adults, infection of the foetus and miscarriage in pregnant women, or neonatal infection [1]. The disease is also associated with a high mortality rate, reaching 20%–30%, and for risk-group patients even 75%. The number of confirmed cases of listeriosis among the inhabitants of the EU demonstrated a growing tendency from 1439 reported in 2005 to 2549 in 2018 [2,3].

Consistent with the increasing trend at European level, an intensification in the occurrence of listeriosis was observed in Marche region, Italy, between January 2015 and May 2015. After that, a total of 24 human cases of listeriosis occurred [4].

During the outbreak a huge number of strains were sent to the Italian National Reference Laboratory for *L. monocytogenes* (It NRL Lm) in order to identify the source of infection, and to carry out trace back and forward activities.

Next generation sequencing of the isolates and comparative genome analysis confirmed a unique strain responsible for the outbreak.

Since 2000, pulsed field gel electrophoresis (PFGE) has become the gold standard for *L. monocytogenes* subtyping and has been extensively used, throughout the world, during outbreak and trace back investigations. Although PFGE is widely accepted and used, in some cases it was found to be inadequate and obsolete [5]. Nowadays, the use of whole genome sequencing (WGS) for the characterization of pathogens has become a standard component of infectious disease surveillance and WGS-based differentiation of *L. monocytogenes* isolates has become a pivotal tool for listeriosis outbreak investigations in the USA [6,7]. Recent advances in sequencing technologies and analysis tools have rapidly increased the output and the analysis speed and also reduced the costs of WGS [8].

Despite this, PFGE, thanks to its internationally accredited and standardized protocol, is often joined with the Single Nucleotide Polymorphism (SNP)-based analyses [6]. In a hypothetical scenario, when outbreak occurs, thousands of samples could be processed and a huge number of *L. monocytogenes* strains can be isolated from human, food processing environment, and food sources leading to a possible bottleneck in both sequencing/data analysis and PFGE typing. This protocol requires up to 40 working days to accomplish strain typing. The response time of the analyses performed can make the difference between the occurrence of new clinical cases or not, and between minor or major economic loss for the food business operators and farms involved. For all these reasons the use of a screening method is a focal point for proficient outbreak management.

The aim of this paper was to describe the application of a specific real-time PCR screening assay properly designed for the rapid identification of strains potentially related to the listeriosis outbreak occurred in Central Italy between 2015 and 2016.

## 2. Materials and Methods

### 2.1. Bacterial Isolates DNA Extraction

All the strains were received as pure culture on sheep blood agar and subjected to DNA extraction. Stock cultures (Microbank™, Pro Lab Diagnostics Inc., Richmond Hill, ON, Canada) were prepared and stored at −80 °C when the strains were not processed immediately.

Cultures identified as *L. monocytogenes* by biochemical methods were grown overnight in sheep blood agar (Microbiol & C. s.n.c., Cagliari, Italy), picked, and dissolved in 300 μL of nuclease-free water (Ambion™, Thermo Fisher Scientific, Waltham, MA, USA). Then, 100 μL of 20 mg/mL lysozyme was added and incubated for 2 h at 56 °C. Finally, 300 μL of the suspension were transferred to the cartridges provided by the Maxwell 16 Cell DNA Purification Kit (Promega, Madison, WI, USA). DNA extraction was accomplished following the manufacturer’s instructions. DNA was quantified using a Qubit dsDNA HS (High Sensitivity) Assay Kit (Invitrogen, Carlsbad, CA, USA) and purity was checked by a Nanodrop ND-1000 spectrophotometer (Thermo Fisher Scientific, Waltham, MA, USA).

### 2.2. Serogroup

Strain characterization was performed with a molecular serogroup-related PCR completed by the detection of *fla*A [9,10].

Briefly, DNA was extracted as described before and reactions were carried out in a Gene Amp PCR System 9700 thermal cycler (Applied Biosystems, Foster City, CA, USA). PCR products were run in 2% (*w*/*v*) agarose gel in 1× tris/borate/EDTA (TBE) buffer (Biorad, Hercules, CA, USA) and visualized by SYBR™ Safe DNA Gel Stain (Thermo Fisher Scientific, Waltham, MA, USA).

### 2.3. PFGE

PFGE analysis was performed, according to PulseNet protocol [11], as described previously [12]. Briefly, the bacterial suspensions were embedded in agarose, lysed, washed, and then digested with the restriction enzymes. The digested samples underwent electrophoresis in 1% (*w*/*v*) SeaKem Gold agarose (Lonza Rockland, Inc., Rockland, ME, USA) in 1 × TBE (Sigma-Aldrich, St. Louis, MO, USA) by using the Chef MapperXA system (Bio-Rad Laboratories, Hercules, CA, USA) at 6 V/cm, with a pulse time between 4 and 40 s for 19 h.

PFGE profiles were analyzed using BioNumerics version 7.5 (Applied Maths, Sint-Martens-Latem, Belgium). The similarities between the *Asc*I and *Apa*I macrorestriction profiles (MRPs) were calculated using the Dice coefficient, applying an optimization coefficient and band tolerance of 1.0% for both enzymes.

### 2.4. Next Generation Sequencing

WGS was used, in the first instance, to find common genomic regions for developing a real-time PCR screening test and then to perform outbreak inclusion/exclusions by SNPs analysis.

The DNA from *L. monocytogenes* strains were sequenced by the NextSeq500 Illumina platform using the Nextera XT protocol. Raw data were trimmed and assembled using Trimmomatic [13] and Spades 3.11 [14], respectively, with default parameters for the Illumina platform 2 × 150 chemistry. Then the genomes annotation was performed by Prokka [15] with default parameters except for the bacterial genetic code (−gcode 11). Annotation data were used to build a pan-genome matrix using roary [16] with default parameters and the .gff files obtained by the annotation.

Genetic relationships among isolates and outbreak inclusion/exclusions were performed by a SNP-based approach, using the reference free tool, kSNP3 [17], and a kmer value of 21 as indicated by Morganti et al. [18]. The core SNPs matrix was used as input to draw a neighbor-joining (NJ) phylogenetic tree using Mega6 [19].

### 2.5. Real-Time PCR

Aligning whole genome sequences of 12 outbreak clinical strains by ClustalW, common genomic sequences were identified [20]. Multiple sequence alignment found 14 highly conserved regions (coding for hypothetical proteins, recombinase family protein, transcriptional regulator, DEAD-DEAH box helicase, GNAT family acetyltransferase, and N-6 DNA methylase).

Primer Express v3.0.1 software (Applied Biosystems, Foster City, CA, USA) was used to design TaqMan assays for all the 14 genome regions. The recombinase family protein gene (rec) and the transcriptional regulator (trans) assays, which showed the best score, were selected to develop a multiplex real-time PCR screening method. The assays were optimized in a duplex real-time PCR using 6-Carboxyfluorescein (FAM) and 6-Carboxy-4′,5′-Dichloro-2′,7′-Dimethoxyfluorescein (JOE) as fluorescent reporter dyes, respectively (Table 1).

Oligonucleotides were synthesized by Eurofins Genomics (Ebersberg, Germany). The 20 μL reaction volume contained 5 μL of purified DNA (2 ng/μL), 10 μL of GoTaq Probe qPCR Master Mix 2×, 300 nM for both Rec forward and reverse primers, 150 nM for Rec probe, 600 nM for both Trans forward and reverse primers, 200 nM for Trans probe, and nuclease-free water up to final volume. Real-time PCR were performed on the 7900 HT Fast Real-Time PCR System (Applied Biosystems, Foster City, CA, USA) using the following thermal profile: DNA polymerase activation at 95 °C for 20 s tailed by 35 cycles of denaturation at 95 °C for 1 s and annealing/extension at 60 °C for 20 s.

Three replicates of five ten-fold DNA serial dilutions from 20 to 0.002 ng/μL were amplified to create the standard curve. The efficiency (E) was calculated according to the formula E = (10^−1/slope^ − 1) × 100 [21].

## 3. Results

Between January 2015 and September 2016, a total number of 37 clinical strains isolated from patients exhibiting listeriosis symptoms and 1374 strains correlated to the outbreak were received by the It NRL Lm. Among them, 1397 were screened with real-time PCR, 1230 were typed with molecular serogroup-related PCR, and 490 with PFGE and WGS.

### 3.1. Workflow

**Figure 1:**
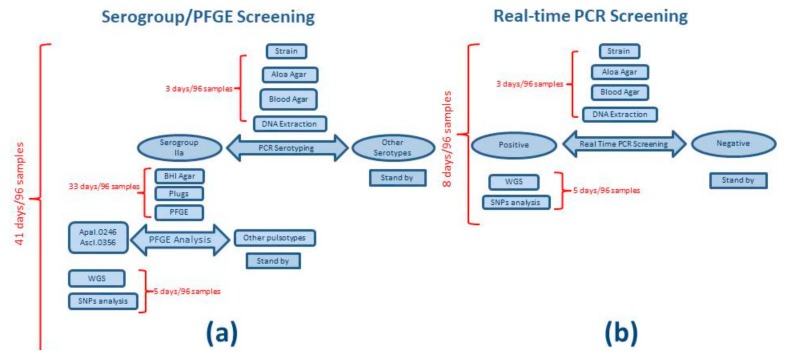
Analysis workflow and turnaround time to analyze 96 samples. Cases (**a**) and (**b**) show time of analysis applying serogroup and PFGE and real-time PCR as screening test to perform outbreak inclusion/exclusion, respectively.

Real-time PCR and serogroup determination were used as alternative screening methods to give priority for deeper typing by Next Generation Sequencing (NGS) and PFGE. Before real-time PCR, strain selection was based on serogroup determination and then PFGE. The robustness coupled with the reduced turnaround time for analysis made the real-time PCR the method of choice for screening purposes. For every sample, up to five colonies were screened and then one PCR-positive colony was sequenced by NGS and genotyped by SNPs analysis (Figure 1). All the colonies sequenced were also genotyped by PFGE.

### 3.2. Serogrouping and PFGE

Serogrouping analysis clustered 1224 isolates into four serogroups (IIa flaA+, IIc flaA−, IVb, and IIb), one strain was associated to serogroup IIc flaA+, and five strains were identified as *Listeria* spp.

The main serogroup was IIa flaA+ (47.7% *n* = 587). Serogroup IIc flaA− was detected in 28.4% of the strains (*n* = 349), while serogroups IIb and IVb were identified in 13.3% (*n* = 164) and 10.1% (*n* = 124), respectively.

All the strains included in the outbreak showed the same serogroup, IIa flaA+, and pulsotype *Apa*I.0246 *Asc*I.0356 as defined by the European Centre for Disease Prevention and Control (ECDC).

**Figure 2:**
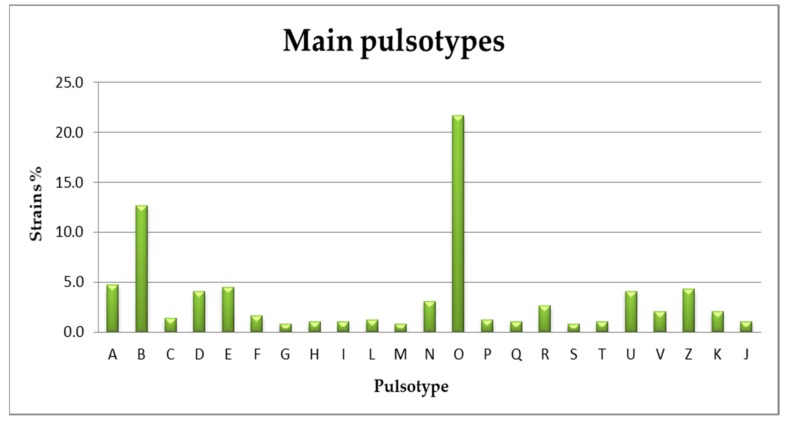
Main pulsotypes detected in 490 *Listeria monocytogenes* strains analyzed during the outbreak investigation.

The PFGE analysis was carried out on 490 strains with both the enzymes and yielded 97 combined pulsotypes in addition to the outbreak profile that represented the prevalent one (21.6%) (Figure 2).

### 3.3. Next Generation Sequencing

DNA of real-time PCR positive strains was sequenced by NGS and then related strains typed by PFGE. The theoretical coverage for each sample was higher than 70× with an average reads quality higher than 32.

**Figure 3:**
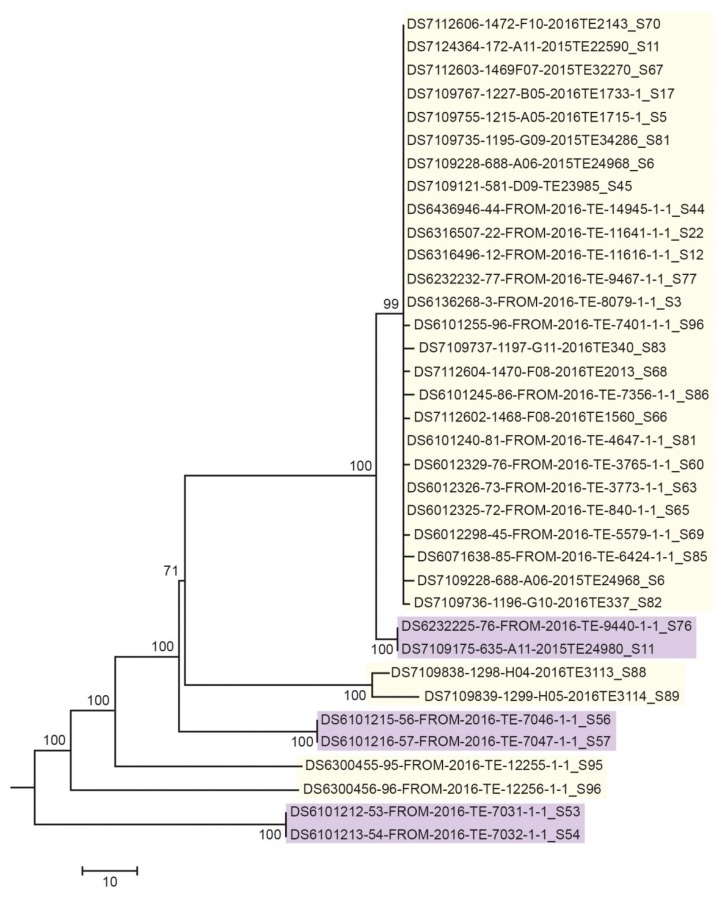
Phylogenetic relationships (neighbor joining (NJ)) among a selected subset of 38 strains analysed during the outbreak investigation. Strains showing pulsotype *Apa*I.0246 *Asc*I.0356 are highlighted in yellow; strains positive to real-time PCR but with a different pulsotype from the outbreak strain are highlighted in purple.

Genetic relationships among isolates were evaluated through a SNP-based approach. The threshold for outbreak inclusion was set to 60 different SNPs. The neighbor joining phylogenetic tree showed a strong correlation between strains included in the outbreak, suggesting a high clonality of the collected strains (Figure 3).

### 3.4. Real-Time PCR

Analytical specificity, the ability of the assay to distinguish the DNA target from non-target DNA, was assessed on 639 strains not included in the outbreak based on SNP analysis, while 111 strains, included in the epidemic cluster, were used as positive controls.

The efficiencies of real-time PCR assays for the target Rec and Trans were 99% and 94%, respectively (Table 2). All the 111 strains previously included in the outbreak by SNP analysis were correctly detected by both assays, while the DNA of the 639 strains not included in the outbreak were not amplified. The analytical specificity calculated was 100% (lower confidence limit 99.5% and upper confidence limit 100%).

The method was then performed on 1397 strains during the outbreak investigation. A total of 1178 (84.3%) strains turned out to be negative, while 219 (15.7%) were detected by both targets. All positive samples were immediately sequenced by NGS and typed by molecular serogroup-related PCR and PFGE to confirm the outbreak group identity. Twenty-three out of 219 strains were classified as PCR false positive by PFGE and SNP analysis of WGS data. Of those 23 strains, 15 strains showed different profiles respect to the outbreak pulsotype (*Apa*I.0246, *Asc*I.0356). The leftover eight strains, despite having the same outbreak pulsotype, were epidemiologically unrelated and showed a number of SNPs above the cut-off (Figure 3).

## 4. Discussion

In Italy, in 2018, notification rate of invasive listeriosis was 0.29 cases per 100,000 population, lower than the average of European countries (0.47). Indeed, despite *Listeria* rarely exceeding the EU food safety limit tested in ready-to-eat food, many European countries reported rates higher than 0.80 (Estonia, Finland, Spain, Sweden, Denmark, Luxemburg, and Germany) [3].

Rapid identification of contaminated food and food processing industries is critical to stop the diffusion of pathogenic strains and minimize the number of cases in a foodborne outbreak. Depending on the setting (local, national, or international), one or more molecular methods needs to be carried out for typing and subtyping the involved strains.

Between January 2015 and February 2016, a total number of 63 *L. monocytogenes* strains isolated from food samples potentially related to the outbreak were sent to the It NRL Lm in order to identify the source of infection. In February 2016, strains isolated from a sample of hog head cheese (a typical pork-derived meat jelly-seasoned product) were identified as serotype 1/2a and showed 100% genetic similarity with the outbreak pulsotype by PFGE [4]. After that, deep tracing back and forward investigations were carried out and 1348 strains were isolated from both contaminated food and food processing environmental samples.

Before the use of the real-time PCR method, isolates were screened by molecular serogrouping. As described in several studies [22,23,24], IIa is the main serogroup within strains isolated from food and food processing environments. Accordingly, in our study almost half of the typed strains were IIa flaA+, thus molecular serogrouping was not suitable for the screening purposes. Moreover, considering the number of strains received at the IT NRL Lm and the need to establish the inclusion or exclusion of food or food processing industries in the outbreak, the usual workflow was no more applicable, because the time needed to obtain results of molecular serogrouping coupled with PFGE analysis became too long.

A PCR-based method was developed to decrease the turnaround time for the screening. The real-time PCR method was able to quickly analyze up to 96 strains in 2–4 h and to identify all the strains potentially related to the outbreak. Positive isolates were then further investigated by PFGE and SNP analysis.

Positive results were also observed in 10.5% of strains not related to the outbreak. However, false positive outcomes are generally accepted for the inclusivity scope of a screening assay, designed to limit the number of the strains addressed for further analysis, but absolutely able to include all the strains potentially related to the outbreak.

Applying the described real-time PCR assay, we were able to drastically decrease time and costs of analysis during the outbreak. The developed strategy selected only 219 out of 587 strains, classified as IIa by molecular serogrouping, which were submitted to SNP analysis and PFGE with priority. As the estimated cost of PFGE per single strain is between 20.9 and 23.3 Euro, depending on the gel size [25], the gain ranged from 7691 and 8574 Euro. Moreover, the rapid response time of the analyses limited economic loss for the food business operators and farms involved. Overall, serogroup/PFGE screening was demonstrated to be less effective than the assay developed in this study.

## 5. Conclusions

WGS data were used as “starting point” to develop a PCR-based diagnostic test. We demonstrated that our novel real-time PCR assay can screen thousands of *L. monocytogenes* strains, significantly reducing time and costs.

Despite the fact that the method was specifically designed for *L. monocytogenes* strains involved in the listeriosis outbreak that occurred in Central Italy, this case report represents a proof of concept suggesting to the scientific community a new approach for any outbreak management.

WGS data were finally used as “closing point” of the workflow, the SNP-based clustering method for *L. monocytogenes* isolates enabled discrimination of strains indistinguishable in PFGE, but not correlated to the outbreak, and identification of the strains isolated from a hog head cheese sample produced by a facility located in Central Italy as the possible source of human infection.

## Figures and Tables

**Table 1:** Primers and probes sequences for Rec and Trans real-time PCR.

Oligonucleotide	Sequence 5′-3′	Size
Rec-fwd	AAATAATGCGGAGTTAAAAGGTGAA	74 bp
Rec-rev	TGGACTGCATTTGGTATGTGAGT	
Rec-probe	FAM-TACGGATTGCCGTCCCCGAAAGT-BHQ1	
Trans-fwd	CTCATTACGTTGATTGGCATACG	79 bp
Trans-rev	GGTTCGTGGTCTCCTTTTACAATAA	
Trans-probe	JOE-AACGAAGAAAAGGGAAAAACTCCCACCC-BHQ1	

**Table 2:** Analytical performance of the duplex real-time PCR method.

Assay	Slope	R^2^	Efficiency
Rec	−3.34	0.998	99%
Trans	−3.47	0.999	94%

Efficiency = (10^−1/slope^ − 1) × 100.
